# DEAD Box Protein DDX1 Regulates Cytoplasmic Localization of KSRP

**DOI:** 10.1371/journal.pone.0073752

**Published:** 2013-09-04

**Authors:** Chu-Fang Chou, Wei-Jye Lin, Chen-Chung Lin, Christian A. Luber, Roseline Godbout, Matthias Mann, Ching-Yi Chen

**Affiliations:** 1 Department of Biochemistry and Molecular Genetics, University of Alabama at Birmingham, Birmingham, Alabama, United States of America; 2 Department of Proteomics and Signal Transduction, Max-Planck Institute of Biochemistry, Martinsried, Germany; 3 Department of Oncology, University of Alberta, Cross Cancer Institute, Edmonton, Alberta, Canada; Colorado State University, United States of America

## Abstract

mRNA decay mediated by the AU-rich elements (AREs) is one of the most studied post-transcriptional mechanisms and is modulated by ARE-binding proteins (ARE-BPs). To understand the regulation of K homology splicing regulatory protein (KSRP), a decay-promoting ARE-BP, we purified KSRP protein complexes and identified an RNA helicase, DDX1. We showed that down-regulation of DDX1 expression elevated cytoplasmic levels of KSRP and facilitated ARE-mediated mRNA decay. Association of KSRP with 14-3-3 proteins, that are predominately located in the cytoplasm, increased upon reduction of DDX1. We also demonstrated that KSRP associated with DDX1 or 14-3-3, but not both. These observations indicate that subcellular localization of KSRP is regulated by competing interactions with DDX1 or 14-3-3.

## Introduction

Inherently unstable mRNAs contain *cis*-acting elements within the 3’ untranslated regions (UTRs) that direct their rapid decay through activation of mRNA decay pathways [1,2]. The AU-rich elements (AREs), prominent instability elements, direct rapid mRNA decay by a process referred to as ARE-mediated mRNA decay (AMD) [3-5]. A number of ARE-binding proteins (ARE-BPs) target ARE-containing mRNAs for decay. By contrast, stabilizing ARE-BPs protect them from decay [6,7]. There are at least six RNA-binding proteins involved in AMD. Zinc finger proteins, tristetraprolin (TTP) and butyrate response factor 1 and 2 (BRF1 and BRF2), are potent stimulators of AMD [8-11]. AU-rich element RNA-binding protein 1 (AUF1) modulates mRNA decay and either stabilizes or destabilizes ARE-containing mRNAs depending on experimental systems [12-15]. KSRP (K homology Splicing Regulatory Protein) is essential for AMD [17-20] and was originally identified as a component of a protein complex that assembles on an intronic *c-src* enhancer required for neuronal-specific splicing [16]. In contrast, Hu antigen R (HuR) stabilizes ARE-containing mRNAs [21].

The current model for AMD is that decay-promoting ARE-BPs recruit the mRNA decay machineries onto the mRNA molecule, thereby triggering its deadenylation, 5’ decapping, and subsequent degradation [18,22,23]. Several lines of evidence have demonstrated that the activities of ARE-BPs are also regulated by additional factors. HuR and AUF1 are predominately localized to the nucleus, but their presence in the cytoplasm is enhanced under stress conditions [24-28]. Furthermore, cytoplasmic localization of TTP and AUF1 is increased by their interactions with 14-3-3 protein family [29,30]. The increased cytoplasmic localization of the p37 AUF1 isoform through interaction with 14-3-3σ enhances the decay of ARE-containing mRNA [30].

KSRP was shown to localize predominately in the nucleus owing to its nuclear localization sequence (NLS) at the N-terminus [31]. However, KSRP accumulates in stress granules (SGs) under oxidative stress [32]. DDX1, a DEAD box protein, and KSRP colocalize in SGs and form a RNA-protein granule complex [33]. KSRP has also been reported to be phosphorylated by AKT and this phosphorylation elevates its interaction with 14-3-3ζ, rendering its restriction to the nucleus [34,35]. These observations indicate that KSRP shuttles between the nucleus and the cytoplasm and its subcellular localization is regulated.

To determine whether the decay-promoting activity of KSRP is controlled by other factors, we purified KSRP-associated complexes and identified several co-purified proteins. One of the proteins was indeed DDX1 and its function in regulating KSRP activity and AMD was investigated. We showed that down-regulation of DDX1 facilitated AMD. We attribute this effect to an increased cytoplasmic KSRP mediated by an elevated interaction with the predominately cytoplasmic 14-3-3 proteins. We also showed that DDX1 competed with 14-3-3 for interaction with KSRP. Our findings indicate that the competing interactions of DDX1 or 14-3-3 with KSRP regulate the cytoplasmic-nuclear shuttling of KSRP, leading to a modulation of its activity in AMD.

## Materials and Methods

### Plasmids

To express TAP-tagged KSRP, the N-terminal TAP tag [36] was amplified by PCR and subcloned into the KpnI and EcoRI sites of pcDNA3-FLAG-KSRP [37]. Plasmids expressing FLAG-tagged KSRP fragments, KH1-4 (amino acids 133-500) and KSRPc (amino acids 501-711), and mRNA reporters, expressing GB-ARE^GMCSF^ and GB-GAPDH, were previously described [23,37]. A plasmid expressing EGFP-KSRP [38] and a plasmid expressing FLAG-DDX1 [39] were also described.

### siRNAs

Sequences of siRNAs against bacterial chloramphenicol acetyltransferase (CAT) and DDX1 are GACGGUGAGCUGGUGAUAU and UGGCAUGGGUGUAGAGCUA, respectively.

### Antibodies

Antibodies against KSRP and HuR [17,18] and polyclonal DDX1 antibodies [40] were previously described. Antibodies against AUF1 and 14-3-3 (H8) were purchased from San Cruz Biotechnology and monoclonal antibodies against FLAG and α-tubulin were purchased from Sigma. Polyclonal antibodies against origin replication complex subunit 2 (ORC2) were kindly provided by Dr. Igor Chesnokov (University of Alabama at Birmingham).

### Purification of KSRP complexes

Human HT1080 fibrosarcoma cells, kindly provided by Dr. Christoph Moroni [41], were transfected with pcDNA-TAP or pcDNA-TAP-KSRP and individual stable transfectants were selected. TAP-KSRP and associated proteins were purified using TAP procedures. Purified proteins were analyzed by mass spectrometry and LC-MS/MS analysis as described [17,42].

### Co-immunoprecipitation assays

Cell extracts were treated with RNase A (0.2 mg/ml at room temperature for 10 min) and incubated with 10 µl (bed volume) of anti-FLAG agarose (Sigma) for 4 hr at 4°C in a buffer containing 50 mM Tris, 150 mM NaCl, and 0.5% NP-40. For DDX1 competition assays, cell extracts containing FLAG-KSRP were incubated with anti-FLAG agarose and GST-DDX1 (purchased from Abnova) or GST. The beads were washed eight times with a buffer containing 50 mM Tris, 150 mM NaCl, and 0.05% NP-40 and immunoprecipitated materials were eluted with 50 µl of FLAG peptide (200 µg/ml; Sigma) for 30 min at 4°C. The eluted fractions were subjected to immunoblot analysis.

### Subcellular fractionation

Cytoplasmic extracts were prepared by incubating cells with 100 µl of hypotonic buffer A (10 mM HEPES [pH 7.9], 10 mM KCl, 1.5 mM MgCl_2_ containing protease inhibitors [2 µg/ml of leupeptin, 2 µg/ml of aprotinin, and 0.5 mM phenylmethylsulfonyl fluoride) on ice for 5 min and lysed by addition of 6.25 µl of 10% NP-40 on ice for additional 5 min. Nuclei were pelleted (10,000 rpm, 5 min, 4°C) and supernatants were saved. The nuclear pellets were washed once with 1 ml of PBS, centrifuged, incubated in extraction buffer C (20 mM HEPES [pH 7.9], 0.45 M NaCl, 1 mM EDTA) on ice for 20 min. Cellular debris was removed by centrifugation (13,000 rpm, 4°C for 5 min) and supernatants were saved. Total extracts were prepared by incubating cells with 100 µl of RIPA buffer (25mM Tris-HCl [pH 7.6], 150mM NaCl, 1% NP-40, 1% sodium deoxycholate, 0.1% SDS) on ice for 10 min.

### Cells, transfection, and mRNA decay assays

HeLa-TO cells (purchased from Clontech) were plated onto six-well plates and transfected with mRNA reporters and siRNA using lipofectamine. To examine mRNA decay, 16 hr after transfection cells were collected and replated onto 35-mm plates. After another 24 hr, the cells were treated with medium containing doxycycline (2 µg/ml) and cytoplasmic RNA was isolated at different times. Reporter mRNA levels were analyzed by Northern blot as described [23,37].

### Fluorescence microscopy

Cells were fixed with 2% cold paraformaldehyde for 20 min followed by incubation with 0.5% Triton X-100 for 10 min at room temperature. After blocking with PBS containing 2% BSA and 5% FBS, cells were incubated with mouse anti-FLAG (1:5000) or mouse anti-KSRP antibodies. Secondary antibodies were conjugated with FITC (Jackson Immunoresearch). Cells were visualized using an Olympus IX70 inverted fluorescence microscope. Images were acquired with a Photometrics 1400 charge-coupled device camera under the control of IPLab software (Scanalytics, Inc.).

## Results and Discussion

**Figure 1:**
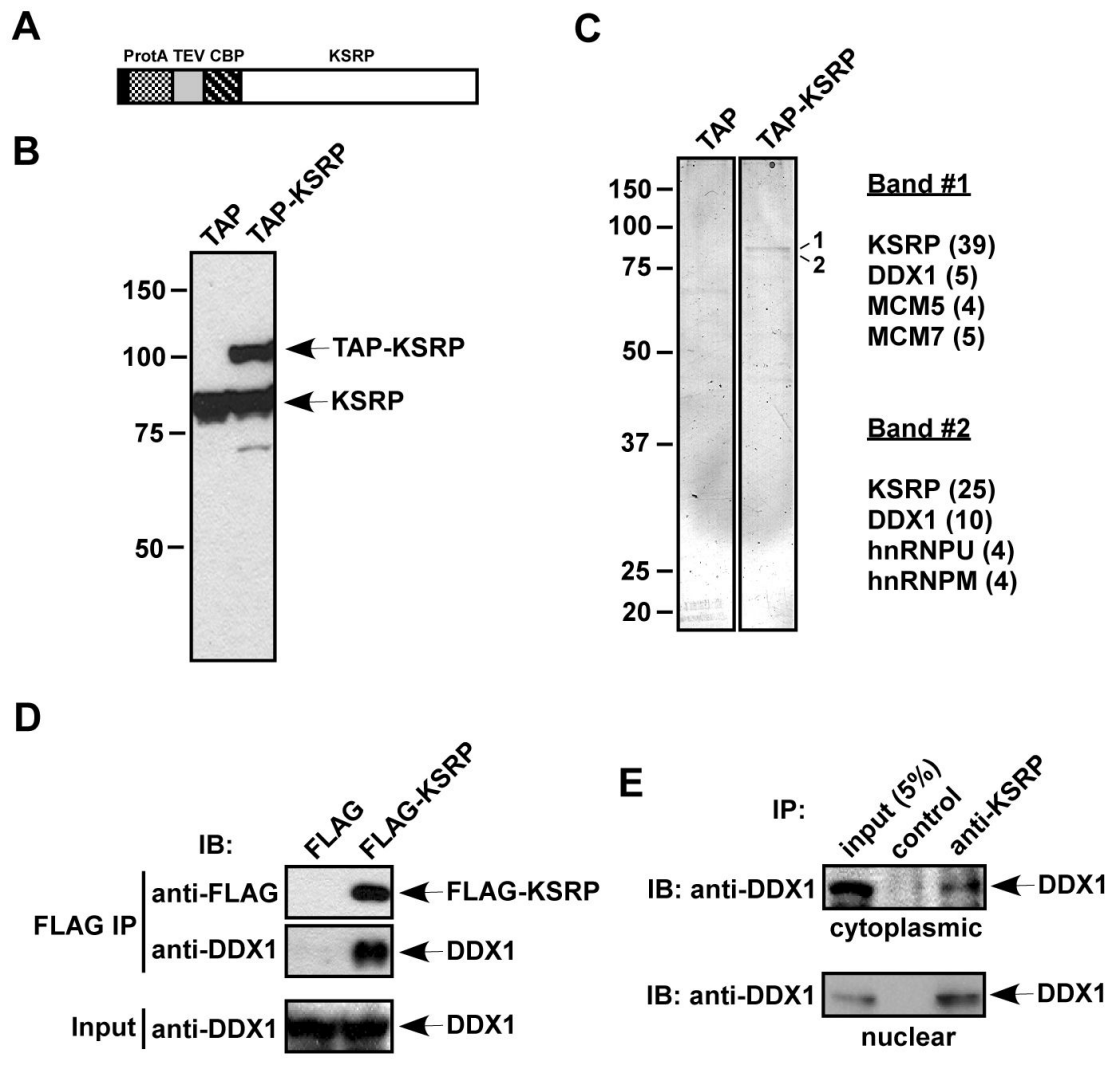
Purification of KSRP-associated proteins. (A) A schematic diagram of TAP-KSRP. Sequences encoding the TAP tag containing protein A (ProtA), a TEV protease cleavage site, and a calmodulin binding peptide (CBP) were fused to the N-terminus of KSRP. (B) Extracts from HT1080 stable cells expressing the TAP tag or TAP-KSRP were analyzed by anti-KSRP immunoblotting. (C) Extracts containing TAP and TAP-KSRP were treated with RNase A and subjected to TAP purification. The purified fractions were analyzed by silver staining. Two bands detected only in the TAP-KSRP fraction are labeled (1 and 2). Proteins identified from bands 1 and 2 by mass spectrometry are indicated. Numbers of observed peptides are denoted in parentheses. (D) FLAG or FLAG-KSRP was expressed in HeLa-TO cells. RNase A-treated extracts were subjected to anti-FLAG immunoprecipitation. The precipitates were analyzed by anti-FLAG or anti-DDX1 immunoblotting. 5% of input used for immunoprecipitation was also analyzed by anti-DDX1. (E) RNAse A-treated cytoplasmic and nuclear extracts were immunoprecipitated with an anti-KSRP monoclonal antibody or a control IgG. The precipitates and 5% of input were analyzed by anti-DDX1 immunoblotting.

To identify proteins that are associated with KSRP and may modulate its activity in AMD, we constructed a plasmid which expresses a TAP (tandem affinity purification) tag [43] at the N-terminus of KSRP (TAP-KSRP; Figure 1A). A stable cell line expressing TAP-KSRP was established in human HT1080 fibrosarcoma cells. Immunoblot analysis showed that expression of TAP-KSRP in these cells was equivalent to that of endogenous KSRP (Figure 1B). Total extracts from control cells, expressing the TAP tag only, and TAP-KSRP-expressing cells were subjected to two steps of affinity chromatography [36]. The eluted proteins were analyzed by SDS-PAGE and silver staining. Two prominent bands (band 1 and band 2) migrated at 80-90 kDa and few very minor bands (which were not readily detectable) were only present in the TAP-KSRP eluate (Figure 1C). The two prominent bands were excised from the gel and analyzed by mass spectrometry. Both band 1 and band 2 consisted of CBP-tagged KSRP, DDX1, and few other proteins, including DNA replication licensing factors (MCM5 and MCM7), hnRNP U, and hnRNP M (Figure 1C). To confirm an association between KSRP and DDX1, we expressed FLAG-KSRP in HeLa cells and performed co-immunoprecipitation assays. DDX1 was found to co-precipitate with FLAG-KSRP (Figure 1D). To test whether endogenous KSRP exists in complex with DDX1 in cell extracts, we immunoprecipitated KSRP from cytoplasmic and nuclear extracts and observed that DDX1 was co-precipitated with KSRP in the cytoplasmic as well as the nuclear fractions (Figure 1E). Thus, we conclude that KSRP and DDX1 exist in a complex in both the cytoplasm and the nucleus.

DDX1 belongs to DEAD box-containing RNA helicase family. This family of proteins is involved in post-transcriptional regulation of gene expression [44]. For instance, DDX1 interacts with the HIV-1 RNA-binding protein, Rev. Rev is predominately localized in the nucleus and escorts unspliced viral mRNAs to the cytoplasm. The down-regulation of DDX1 increases cytoplasmic Rev [45]. We thus focused on investigating the role of DDX1 in controlling KSRP function and AMD.

**Figure 2:**
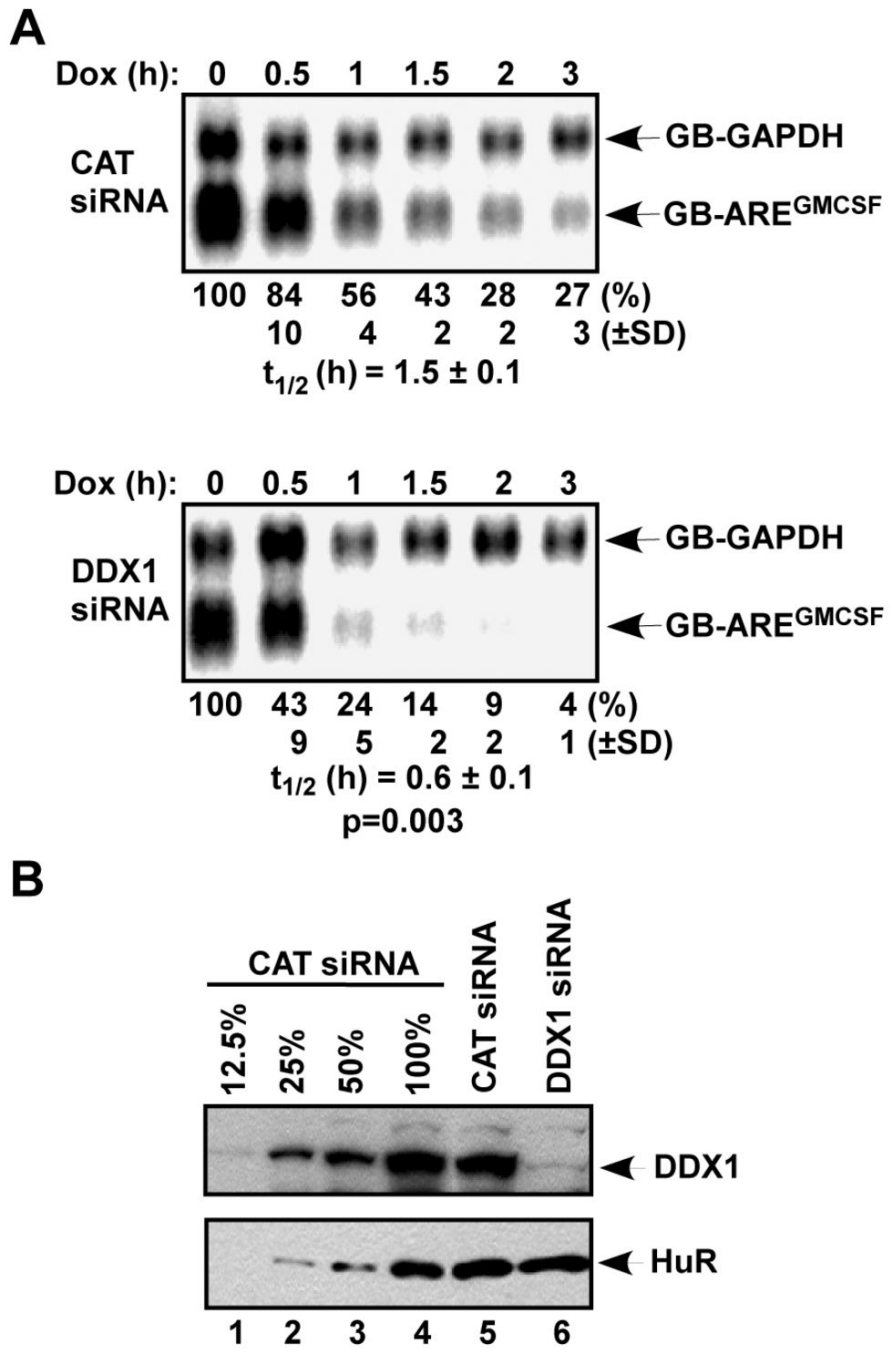
Down-regulation of DDX1 facilitates AMD. (A) HeLa-TO cells were transfected with a construct expressing GB-ARE^GMCSF^ mRNA under the control of a Tet-regulatory promoter and a construct constitutively expressing GB-GAPDH mRNA under the control of the CMV promoter. The cultures were also transfected a control siRNA (CAT) or a DDX1 siRNA. Cytoplasmic RNA was isolated at different time points after the addition of doxycycline (Dox). The levels of GB-ARE^GMCSF^ and GB-GAPDH mRNAs were analyzed by Northern blot. Signals of GB-ARE^GMCSF^ mRNA were quantified by a phosphorimager and normalized to that of GB-GAPDH mRNA. The calculated half-lives (t_1/2_; n=3) of GB-ARE^GMCSF^ mRNA are shown as mean values ± standard deviations from three independent experiments. P value is indicated and calculated by Student’s t-test using Microsoft Excel software. (B) Downregulation of DDX1 by siRNA. Extracts of HeLa-TO cells in (A) were subjected to immunoblot analysis with anti-DDX1 or anti-HuR antibodies. Different amounts of CAT siRNA-treated extracts (12, 25, 50, or 100% of the amounts used in lane 5) were loaded to estimate knockdown efficiency.

First, we examined the effect of DDX1 knockdown with an siRNA on the decay of an ARE-mRNA. A globin mRNA reporter containing the 3’ UTR of GM-CSF (GB-ARE^GMCSF^) was expressed in HeLa-Tet Off (TO) cells. The decay of the reporter mRNA was monitored in a transcriptional pulse-chase assay. While the GB-ARE^GMCSF^ mRNA was degraded with a half-life (t_1/2_) of 1.5 h in control cells, DDX1 knockdown increased the rate of decay by 2-fold (t_1/2_=0.6 h; Figure 2A). These results demonstrate that down-regulation of DDX1 facilitates AMD.

**Figure 3:**
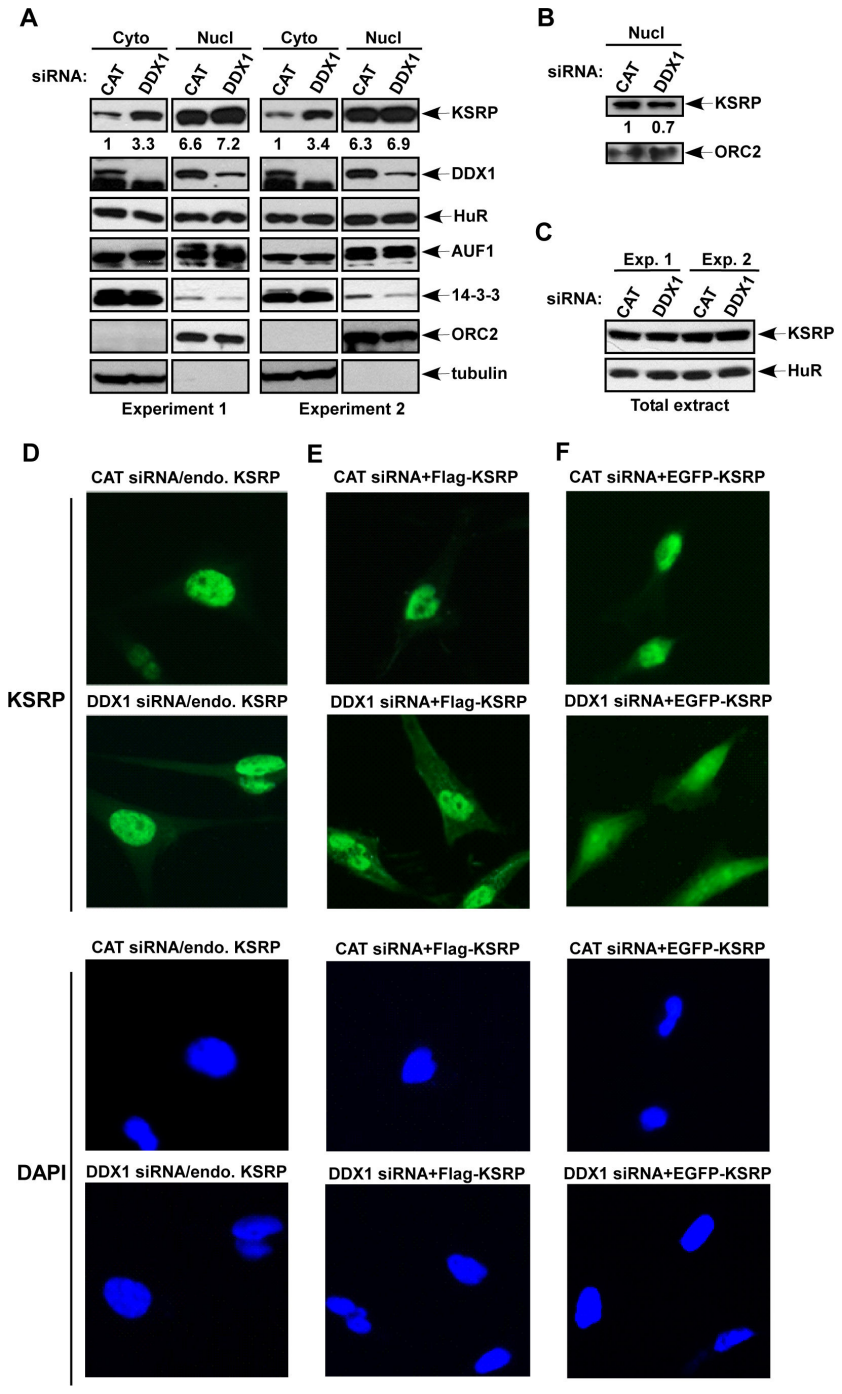
Subcellular localization of KSRP is regulated by DDX1. (A) HeLa-TO cells were transfected with a control siRNA or a DDX1 siRNA. Cytoplasmic and nuclear extracts from equal number of cells were subjected to immunoblot analysis with anti-KSRP, anti-DDX1, anti-HuR, anti-AUF1, or anti-14-3-3 which recognizes all isoforms. Antibodies against cytoplasmic α-tubulin and a nuclear protein, origin recognition complex subunit 2 (ORC2), were also used as controls for subcellular fractionation. Two independent transfection experiments were carried out. Quantification of KSRP levels in the cytoplasmic and nuclear fractions is indicated. (B) The nuclear extracts used in (A) were diluted 5-fold and subjected to immunoblot analysis with anti-KSRP and anti-ORC2. Quantification of the nuclear KSRP levels is indicated. (C) Total extracts of cells transfected with CAT or DDX1 siRNAs were analyzed by anti-KSRP or anti-HuR (D to F). HeLa-TO cells were transfected with CAT siRNA or DDX1 siRNA (D), CAT siRNA or DDX1 siRNA and a construct expressing FLAG-KSRP (E), or CAT siRNA or DDX1 siRNA and a construct expressing EGFP-KSRP (F). Transfected cells were analyzed by anti-KSRP (D), anti-FLAG (E), or GFP signal (F) (top two rows), and by DAPI staining (bottom two rows).

Next, we examined the effect of DDX1 down-regulation on subcellular localization of KSRP. The nuclear and cytoplasmic fractions were separately collected. As shown in Figure 3A, the levels of nuclear KSRP were more than 6 times higher than those in the cytoplasm, and DDX1 was present in the cytoplasmic as well as nuclear fractions with a higher nuclear level. Down-regulation of DDX1 resulted in a 3 fold increase in cytoplasmic KSRP, but the levels of nuclear KSRP were not altered compared with control cells. By contrast, cytoplasmic levels of HuR and AUF1 were not altered in cells treated with DDX1 siRNA compared to that treated with a control siRNA (Figure 3A). The lack of detectable reduction in nuclear KSRP levels was likely due to overloading of the nuclear extracts. To test this possibility, we reduced the amounts of nuclear extracts (5 fold dilution) and observed a 30% reduction in nuclear KSRP upon down-regulation of DDX1 (Figure 3B). The increase in KSRP levels in the cytoplasm was not due to an elevation of KSRP expression, as the total protein level was not altered in DDX1 siRNA-treated cells as compared to that in control siRNA-treated cells (Figure 3C).

To further confirm the elevation of KSRP in the cytoplasm, subcellular localization of endogenous KSRP was assessed by immunofluorescence analysis. While the staining of KSRP in the cytoplasm was barely detected in control siRNA-treated cells, down-regulation of DDX1 significantly increased the cytoplasmic staining (Figure 3D). Similar results were observed by using transiently transfected epitope-tagged KSRP, including FLAG-KSRP (Figure 3E) and EGFP-KSRP (Figure 3F). Altogether, these data indicate that the subcellular localization of KSRP is regulated by DDX1 and suggest that the increased cytoplasmic KSRP likely leads to enhanced AMD.

**Figure 4:**
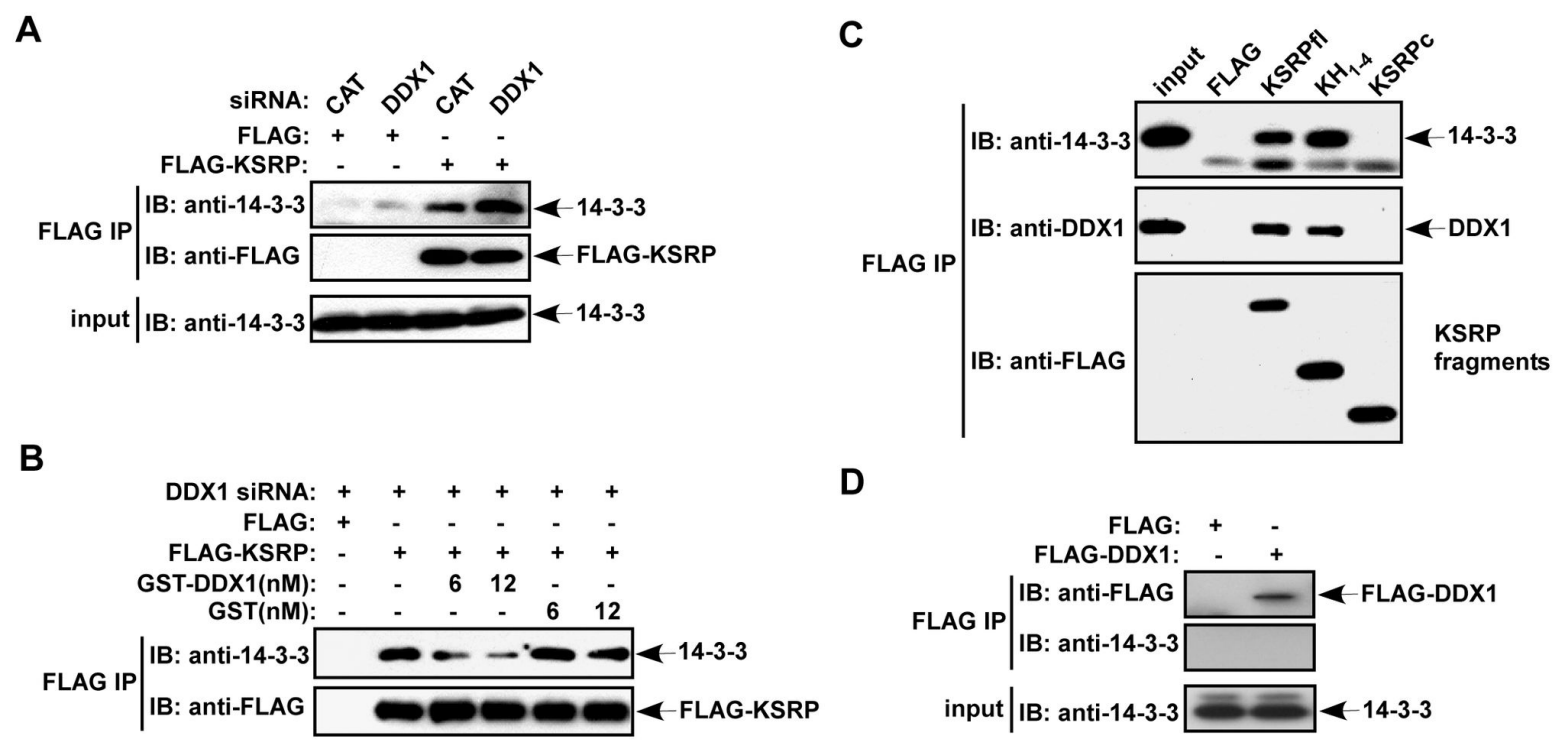
DDX1 interferes with KSRP association with 14-3-3 and competes with 14-3-3 for interaction with KSRP. (A) HeLa-TO cells were cotransfected with a FLAG-KSRP expression vector or a control FLAG vector and a control siRNA or a DDX1 siRNA. Cytoplasmic extracts were subjected to anti-FLAG immunoprecipitation. The precipitates were analyzed by anti-14-3-3 and anti-FLAG immunoblotting. 5% input was also analyzed by anti-14-3-3. (B) HeLa-TO cells were cotransfected with a FLAG-KSRP expression vector or a control FLAG vector and DDX1 siRNA. Cytoplasmic extracts were subjected to anti-FLAG immunoprecipitation with the addition of two different concentrations (6 and 12 nM) of recombinant GST-DDX1 or GST. The precipitates were analyzed by anti-14-3-3 and anti-FLAG immunoblotting. (C) HeLa-TO cells were transfected with vectors expressing FLAG-KSRP or FLAG-KSRP fragments consisting of the four KH motifs or the C-terminus. The FLAG immunoprecipitations were subjected to immunoblotting with anti-14-3-3, anti-DDX1, and anti-FLAG. (D) HeLa-TO cells were transfected with a control FLAG vector or a FLAG-DDX1 expression vector. Cytoplasmic extracts were subjected to anti-FLAG immunoprecipitation. The precipitates were analyzed by anti-14-3-3 and anti-FLAG immunoblotting. 5% input was also analyzed by anti-14-3-3.

Interactions of specific 14-3-3 isoforms with TTP and AUF1 are known to render their cytoplasmic dominance [29,30]. To investigate the mechanism that results in elevated cytoplasmic KSRP levels upon DDX1 down-regulation, we examined KSRP association with 14-3-3 in the cytoplasm. Cytoplasmic FLAG-KSRP was immunoprecipitated from extracts of cells treated with either control siRNA or DDX1 siRNA. The amounts of 14-3-3 co-precipitated with FLAG-KSRP were analyzed by immunoblot analysis using an antibody against all 14-3-3 isoforms. Moderate amounts of 14-3-3 were co-precipitated with FLAG-KSRP from extracts of control cells whereas the amounts of 14-3-3 co-precipitated were increased (2-3 fold) in DDX1 siRNA-treated cells (Figure 4A). While we observed an increase in cytoplasmic KSRP levels upon down-regulation of DDX1 (Figure 3A), we did not detect an increase in the amounts of immunoprecipitated KSRP upon DDX1 knockdown (Figure 4A), suggesting that the immunoprecipitation was saturated. As equivalent amounts of FLAG-KSRP were precipitated between CAT siRNA-treated and DDX1 siRNA-treated cells, the results indicate that a reduction of DDX1 increases the association between KSRP and 14-3-3 in the cytoplasm.

To determine the role of DDX1 in controlling the interaction between KSRP and 14-3-3, we performed co-immunoprecipitation assays after incubating a recombinant DDX1 with cell extracts containing FLAG-KSRP. Addition of GST-DDX1 reduced the amounts of 14-3-3 co-precipitated with FLAG-KSRP as compared with the incubation with GST (Figure 4B). We next examined the regions of KSRP responsible for interactions with 14-3-3 and DDX1. Our results showed that the central RNA-binding domain consisting of the four KH motifs was necessary and sufficient (Figure 4C). Collectively, we suggest that down-regulation of DDX1 elevates the cytoplasmic levels of KSRP through increased interaction with 14-3-3 in the cytoplasm and that DDX1 competes with 14-3-3 for association with KSRP; both interactions require the same or overlapping KSRP domains. Consistent with this hypothesis, 14-3-3 predominantly localized in the cytoplasm (Figure 3A) and DDX1 did not form a complex with 14-3-3 (Figure 4D).

In this study, we showed that subcellular localization of KSRP is regulated by DDX1, which may either enhance the nuclear localization of or reduce the nuclear export of KSRP.

Our data indicate that DDX1 prevents 14-3-3 from interacting with KSRP and the elevated interaction with 14-3-3 upon DDX1 reduction results in an increase in cytoplasmic KSRP where it is retained by association with 14-3-3. This modulation of KSRP subcellular localization then leads to enhanced AMD. However, the mechanism of action through which DDX1 regulates nuclear-cytoplasmic shuttling of KSRP is currently not known. As DDX1 forms a complex with KSRP in the cytoplasm and nucleus, it is likely that DDX1 retains KSRP in the nucleus and/or transports KSRP back to the nucleus. Nevertheless, our data are consistent with previous studies that interactions of TTP and AUF1 with 14-3-3 increase their cytoplasmic levels and overexpression of 14-3-3 facilitates AMD due to elevated cytoplasmic AUF1 [29,30]. Our data contradict a recent study that insulin stimulation or AKT activation increases interaction of KSRP with 14-3-3ζ, which renders KSRP restriction in the nucleus [34,35]. The discrepancy between the report and our study could be attributed to the usage of different cell systems or different isoforms of 14-3-3 that regulate KSRP in different manner.
